# Global Energy Metabolism Deficit in Alzheimer Disease Brain

**DOI:** 10.14283/jpad.2023.91

**Published:** 2024

**Authors:** V. Patel, J. Mill, O.C. Okonkwo, S. Salamat, L. Li, T. Raife

**Affiliations:** 1. Department of Pathology and Laboratory Medicine, University of California-Davis, Sacramento, CA, 95817 United States;; 2. Department of Chemistry, University of Wisconsin-Madison, Madison, WI 53706 United States;; 3. Clinical Science Center, Wisconsin Alzheimer’s Disease Research Center, University of Wisconsin School of Medicine and Public Health, Madison, WI 53792 United States;; 4. School of Pharmacy, University of Wisconsin-Madison, Madison, WI 53705 United States;; 5. Department of Pathology and Laboratory Medicine, University of Wisconsin School of Medicine and Public Health, Madison, WI 53705 United States;; 6. Department of Neurosurgery, University of Wisconsin School of Medicine and Public Health, Madison, WI 53705 United States;

**Keywords:** Alzheimer disease, metabolomics, energy metabolism deficits, ketone body, beta-hydroxybutyrate

## Abstract

**BACKGROUND:**

The understanding of Alzheimer’s disease (AD) has been dominated by the amyloid hypothesis. However, therapies targeting beta-amyloid have largely failed, generating interest in other potential pathogenic factors including energy metabolism.

**OBJECTIVES:**

To interrogate canonical energy metabolism pathways from human prefrontal cortical tissue samples obtained from necropsy comparing AD and control.

**DESIGN, SETTING, AND PARTICIPANTS:**

Postmortem pre-frontal cortical tissue from 10 subjects histologically diagnosed with AD and 10 control (CTRL) subjects was subjected to untargeted metabolomics to interrogate energy metabolism pathways. The samples were matched by age, sex, and postmortem interval.

**METABOLITE MEASUREMENTS:**

Untargeted metabolomics analyses were via Metabolon^®^.

**RESULTS:**

Glucose-derived energy metabolites in the glycolytic and pentose phosphate pathway and the ketone body β-hydroxybutyrate were uniformly decreased in AD brain vs. CTRL brain.

**CONCLUSION:**

This pilot study aimed to identify energy metabolism abnormalities using untargeted brain metabolomics in two independent subject cohorts. Our study revealed a pattern of global energy deficit in AD brain, supporting a growing body of evidence of deficient energy metabolism in AD.

## Introduction

Alzheimer disease (AD) is the most common cause of dementia, accounting for between 60 and 80% of all dementia cases (1). More than six million Americans are currently living with AD, a number that is projected to more than double in the next 40 years (1). There is no cure for AD and despite substantial research only one treatment has been FDA-approved to slow disease progression.

Since the early 1990s, AD research has been dominated by the amyloid hypothesis, a pathogenic model that attributes dysfunction in beta-amyloid production, accumulation, or disposal as the primary cause of AD (2). Beta-amyloid is a sticky protein fragment that accumulates in brain and forms plaques that are a noted characteristic of AD. But, it is not known whether beta-amyloid plaque is a cause or result of neurodegeneration (3). The amyloid hypothesis posits that they are causal, suggesting that plaques disrupt communication between brain cells ultimately leading to cell death. The discovery of gene mutations related to familial AD that cause production of beta-amyloid variants that clump easily support the amyloid hypothesis (4, 5).

Despite decades of research on the amyloid hypothesis, few significant advances have been made in finding a treatment indicating that there may be critical factors missing in the understanding of AD pathology. Healthy individuals with normal cognition can have plaques, and studies have found only weak correlations between plaque density and extent of neurodegeneration (6, 7). Perhaps the strongest evidence of the limitations of the amyloid hypothesis is the lack of success in clinical trials of amyloid-targeting treatments (8). While many potential reasons are cited, including the difficulty of targeting AD in early stages when treatment may be most effective, continued failures have cast doubt on the primacy of the amyloid hypothesis (9). Even the recent success of the beta amyloid-targeting drug aducanumab was met with mixed reactions, with experts divided on whether the small benefit outweighs the cost and potential risks (10). Lecanemab, another monoclonal antibody targeting beta-amyloid plaques, showed reduction in beta-amyloid plaque burden in a subset of patients and received accelerated approval by the FDA for use in patients with mild cognitive impairment or mild dementia. It is also not without significant risks, as amyloid related imaging abnormalities have been reported, necessitating imaging follow up for patients taking the drug (11).

There is a growing consensus that regardless of whether the amyloid hypothesis is correct, there is a need for expanded understanding of AD pathogenesis and additional targets for prevention and treatment. Exploration of fundamental biochemical pathways is a promising area of AD research. Although not fully understood, metabolic changes, such as alterations in energy metabolism and mitochondrial oxidative activity, are noted facets of AD (12, 13). Here we report untargeted metabolomic analyses comparing postmortem AD and control (CTRL) brain with a focus on differences in energy metabolism.

## Methods

### Participants brain samples and subject demographics

Postmortem pre-frontal cortex brain tissue (1.6 gm mean) was collected and frozen from subjects with a clinical diagnosis of AD and subjects without a clinical diagnosis of AD from the University of Wisconsin Alzheimer’s Disease Research Center (ADRC). Pre-frontal cortex was selected as representative of AD pathology when present. The postmortem diagnosis of AD was established by histologic documentation of AD pathology using the NIA-AA criteria. Ten subjects with an NIA-AA score of A3B3C3 (i.e., “high”) AD pathologic change were included in the AD group. The control group (CTRL) consisted of ten subjects without pathologic changes of AD; subjects with a diagnosis of “low” or “intermediate” AD change based on NIA-AA criteria were excluded from the CTRL group. Brain tissue was stored in a −70°C freezer until analyzed. The AD subjects were matched with the CTRL subjects for age, gender, and postmortem interval (PMI). The postmortem neuropathologic diagnoses and demographic information for each subject is listed in Table 1.

### Materials and reagents, sample preparation, and liquid chromatography/mass spectrometry (LC/MS) parameters

Samples were analyzed by Metabolon (Durham, NC). Samples were processed using the automated MicroLab^®^ STAR^™^ system from Hamilton Company (Reno, NV), with several recovery standards added prior to the extraction process for quality control. Proteins were precipitated with methanol then centrifuged. The resulting extract was divided into five fractions for LC-MS analysis. All analyses utilized a Waters ACQUITY ultra-performance liquid chromatograph (UPLC) and a Thermo Scientific Q-Exactive high resolution/accurate mass spectrometer interfaced with a heated electrospray ionization (HESI-II) source and Orbitrap mass analyzer at 35,000 mass resolution. Four UPLC/MS/MS methods were used to identify as many metabolites as possible: one using reversed phase chromatography (RPC) and positive electrospray ionization (ESI+), optimized for hydrophilic compounds; one using RPC and ESI+, optimized for hydrophobic compounds; one with RPC and ESI- and one using hydrophilic interaction chromatography (HILIC) and ESI-. Full details of the LC-MS methods and quality assurance/control protocols can be found in Mill et al. (14). Supplementary Tables 1, 2, and 3 provide details regarding materials, reagents, quality control, and mass spectrometry parameters, respectively.

### Statistical analysis

Raw metabolite concentrations from Metabolon were log transformed for statistical analyses using XLSTAT, PRISM and for the generation of the volcano plot with a fold change threshold at 1.5 and p-value of 0.05. The raw metabolite concentrations were median scaled for analyses.

## Results

**Figure 1.**
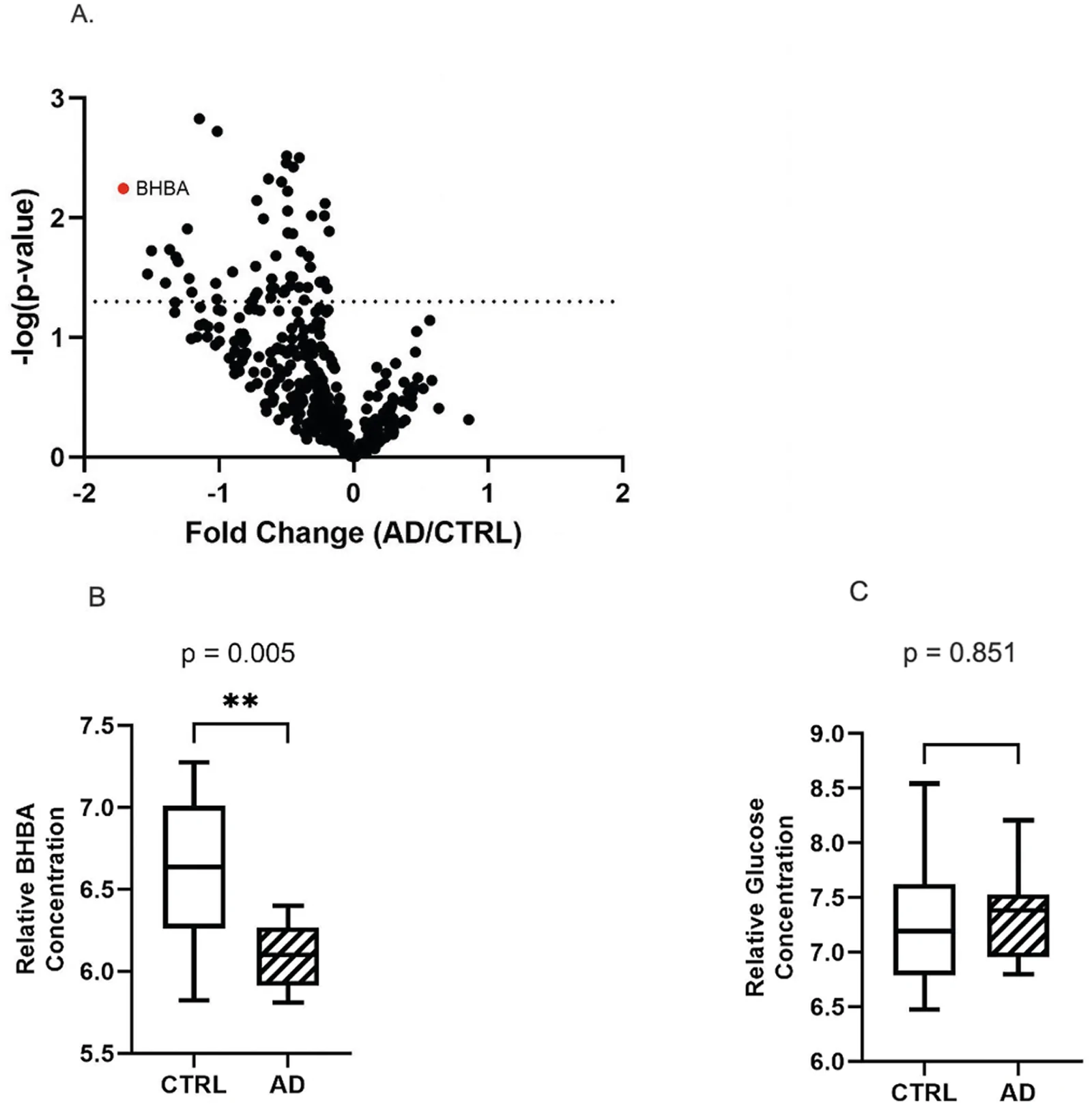
Volcano plot of brain metabolites and BHBA brain concentration. (A) Volcano plot of metabolites in brain showing fold change in AD vs. CTRL subjects and statistically significant metabolites. BHBA was significant (p < 0.005) and decreased in the AD subjects compared to CTRL. Metabolites with a negative value on the x-axis are decreased in brain of AD vs. CTRL, whereas those with a positive value are increased in brain of AD vs. CTRL. All metabolites above the dotted line are statistically significant (p ≤ 0.05). (B) Box plot of log2 transformed brain BHBA concentration showed significantly decreased BHBA concentration in AD subjects. (C) Box plot of log2 transformed brain glucose relative concentration showing no significant difference (p = 0.851) between the two cohorts

**Figure 2.**
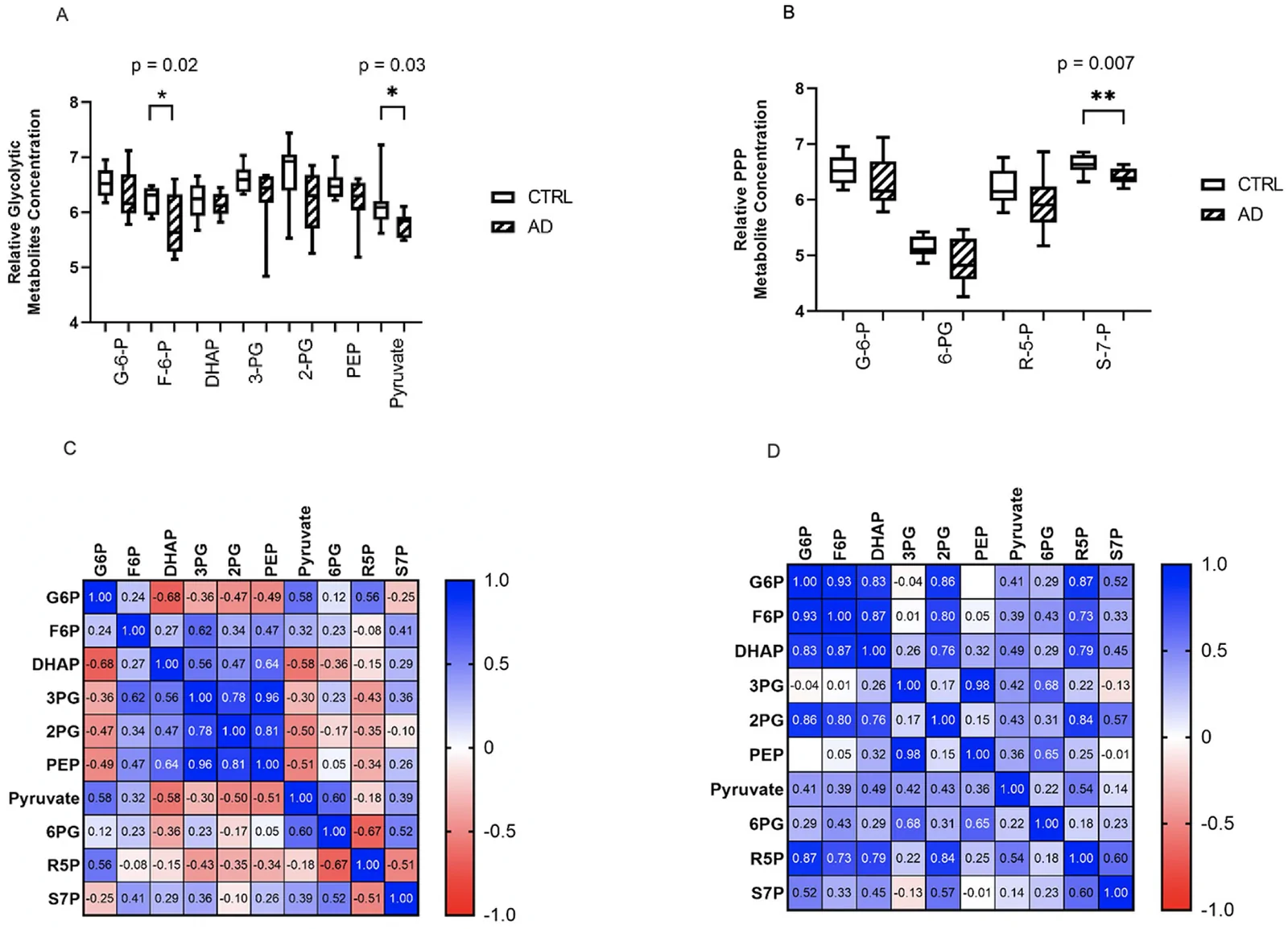
Relative metabolite concentrations of glycolytic and PPP metabolites in brain. Box plots of log2 transformed brain glycolytic (A) and PPP (B) relative metabolite concentrations show a pattern of universally decreased metabolite concentrations in AD compared to CTRL subjects. (C) There are positive correlations among downstream intermediates in the mid-portion of the glycolytic pathway in brain of CTRL subjects, which are negatively correlated with G6P, pyruvate and R5P. (D) In AD, all glucose-derived, nominally correlated metabolites were positively correlated in both glycolysis and PPP (B) Abbreviation: G6P, glucose 6-phosphate; F6P, fructose 6-phosphate; DHAP, dihydroxyacetone phosphate; G3P, glyceraldehyde 3-phosphate; 3PG, 3-phosphoglycerate; 2PG, 2-phosphoglycerate; PEP, phosphoenolpyruvate; 6PG, 6-phosphogluconate; R5P, ribose 5-phosphate; S7P, sedoheptulose 7-phosphate.

Of 590 named biochemicals detected in brain tissue, 65 were significantly different in relative concentration between CTRL and AD specimens (t-test p < 0.05). All intermediate metabolites in the glycolytic pathway were detected except fructose-1,6-bisphosphate and glyceraldehyde 3-phosphate (G3P). The PMI between the CTRL and AD group was not significantly different (p=0.98). Metabolites in a variety of biochemical pathways, including energy metabolism, lipid metabolism, amino acid metabolism, and sphingolipid metabolism showed significant differences between AD and CTRL brain (supplementary Table 4). As reported previously (15), the ketone body β-hydroxybutyrate (BHBA) was markedly decreased in AD brain (p < 0.006) (Figure 1A, 1B). The relative concentration of glucose was increased in AD brain, but this increase was not statistically significant (Figure 1C). The relative concentrations of glucose-derived metabolites in the glycolytic pathway were uniformly decreased in AD vs. CTRL brain, with fructose-6-phosphate (F6P) and pyruvate showing statistical significance (p = 0.02, and p = 0.03, respectively) (Figure 2A). Excluding glucose and lactate, the mean concentration of all combined glycolytic intermediates from G6P to pyruvate was decreased in AD vs. CTRL (p < 0.02). However, glucose was not significantly different between AD and CTRL (p = 0.8510), indicating that decreased glycolytic intermediates were not the result of decreased glucose availability.

Glucose-derived metabolites in the pentose phosphate pathway (PPP) were also uniformly decreased compared to CTRL (Figure 2B). As we previously reported in erythrocytes (16), there was a pattern of positive correlations between glycolytic intermediate metabolites and PPP metabolites. However, in CTRL brain G6P and pyruvate were negatively correlated with intervening glycolytic intermediate metabolites (Figure 2C), which was distinctly different from correlations in AD brain (Figure 2D), suggesting differential trafficking of glucose-derived metabolites in CTRL and AD brain.

**Figure 3.**
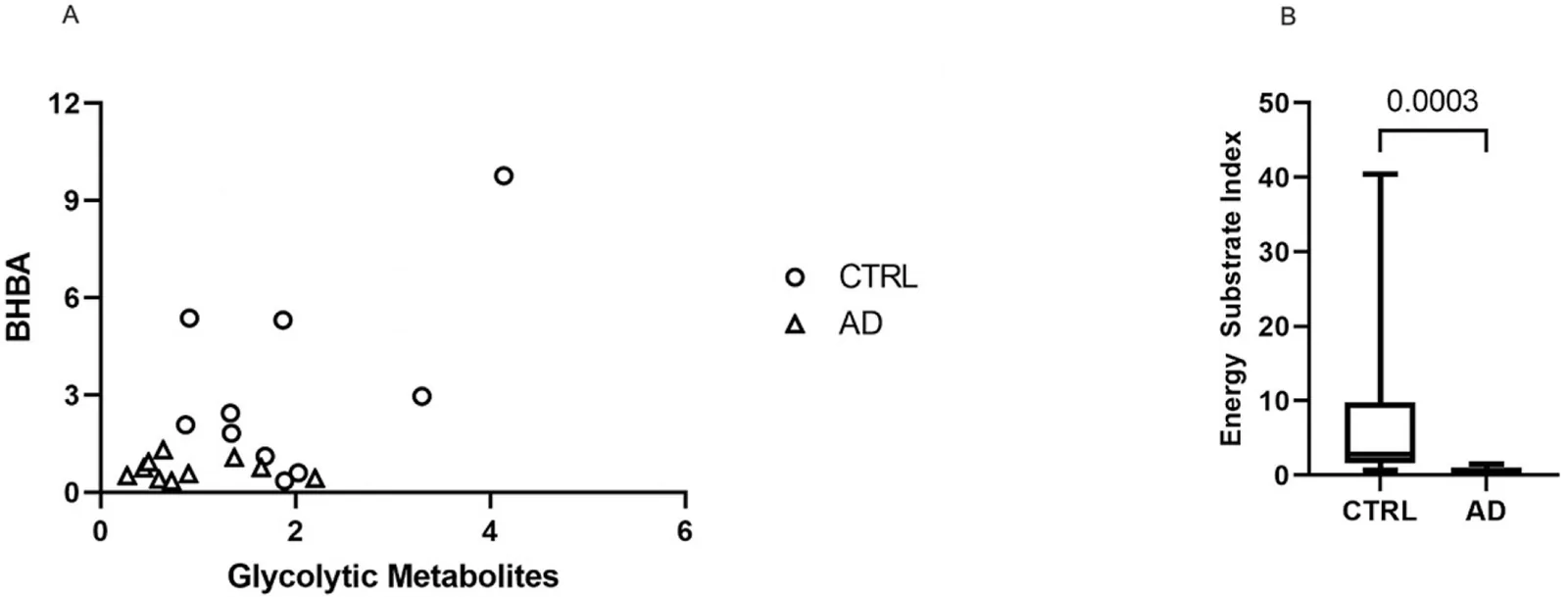
Relative concentration of global energy metabolites in CTRL vs. AD brain. (A) Scatter plot of the mean relative glycolytic and BHBA median scaled* metabolites showing generally higher glycolytic and BHBA metabolite concentrations in CTRL vs. AD. (B) Boxplot of energy substrate index (ESI)** showing a significant (p=0.0003), overall increased composite energy metabolite concentration in CTRL vs. AD (B) *Median scaled values were obtained by calculating the median of the raw value of metabolite concentrations for each metabolite for all 20 subjects. The raw metabolite value for each subject was divided by the median to obtain a median scaled value for each subject. To obtain the mean of the median scaled concentration of glycolytic metabolites, median scaled values for glycolytic metabolites from glucose to lactose were averaged for each of the 20 subjects. The same approach was used to calculate median scaled values of BHBA. **Energy substrate index was calculated by multiplying the mean of the median-scaled glycolytic metabolites by the mean of the median-scaled BHBA metabolite concentration for each of 10 CTRL and 10 AD subjects.

We compared overall energy metabolite availability by correlating the mean concentrations of glycolytic intermediate metabolites with BHBA. The AD cohort is clustered in the lower end of relative concentrations of both glycolytic intermediates and BHBA compared to CTRL (Figure 3A). To derive a total energy substrate index (ESI), we multiplied the mean of the glycolytic intermediate concentrations by the BHBA concentration for each subject. The ESI was significantly decreased in AD brain (p = 0.0003), consistent with a deficit in total energy substrate availability (Figure 3B).

## Discussion

Our study revealed significant differences in concentrations of brain metabolites involving glucose, lipid, amino acid, and ketone body metabolism. Focusing on energy metabolism, in AD brain we observed uniform deficits in glucose-derived metabolite concentrations concurrent with marked deficits in BHBA concentration, indicating a global deficit of energy metabolism. Despite similar concentrations of glucose in CTRL and AD brain, AD glycolytic intermediates from G6P to pyruvate were uniformly decreased, indicating that AD brain is relatively less able to utilize glucose for energy metabolism. Our findings regarding glucose metabolism are concordant with FDG-PET imaging studies showing deficits of glucose uptake and phosphorylation in affected regions of AD brain [17, 18] and the observation that the glucose transporter GLUT3 is decreased in AD brain (19–21).

The brain utilizes glucose as a primary energy substrate through canonical pathways such as glycolysis, the PPP, and oxidative phosphorylation. However, in metabolic states where glucose availability is lacking, the brain utilizes ketone bodies, primarily BHBA and acetoacetate. Ketone bodies are produced in hepatocyte mitochondria, enter the systemic circulation, and are metabolized in the tricarboxylic acid cycle in a variety of tissues including brain. When blood glucose levels are low, hepatic glycogen stores are depleted and the glucagon/insulin ratio is elevated. Energy metabolism shifts to gluconeogenesis, fatty acid degradation, and ketone body production. In AD brain, where we observed a relative decrease in flux through glycolysis and the PPP, the brain would be expected to utilize BHBA as an alternative energy substrate. Paradoxically, we observed a marked decrease in brain BHBA concentrations in AD vs. CTRL. Together, these results suggest that AD brain is less able to efficiently utilize glucose and has less BHBA available for energy metabolism.

In previous work we showed that infusion of BHBA into beta-amyloid Alzheimer model 5XFAD mice decreased beta-amyloid plaque production via inhibition of the inflammasome complex (15). These observations suggest that BHBA may have anti-inflammatory, anti-amyloidogenic physiological roles aside from energy metabolism. Investigations of ketone diet/supplementation in both animal models and human studies have shown cognitive benefits, including in early/pre-AD clinical states (22–27), suggesting a possible neuroprotective role for BHBA. Since BHBA is produced in hepatocytes, the marked decrease in relative BHBA concentrations detected in AD brain vs. CTRL in our studies suggests a possible metabolic link between the brain and liver. Additional studies are needed to determine the cause of decreased BHBA production in the liver of AD subjects and whether this decrease is present in early AD or in mild cognitive impairment.

As previously reported, BHBA concentrations are also significantly decreased in the erythrocytes of AD compared to CTRL subjects, and there is a strong correlation (R2 = 0.9353) between BHBA concentrations in erythrocytes and brain (14). Blood BHBA concentrations therefore appear to accurately reflect brain concentrations and represent a possible biomarker in AD.

In addition to glycolytic intermediates, glucose derived energy metabolites in the PPP were decreased in AD brain. The PPP has two major functions. First, it produces nicotinamide adenine dinucleotide phosphate (NADPH), a molecule critical to maintaining cellular integrity through prevention of oxidative stress via the glutathione pathway. NADPH also provides reducing equivalents for the biosynthesis of macromolecules, such as fatty acids for myelin synthesis, neurotransmitter turnover, and maintenance of redox homeostasis in neurons. Second, the PPP provides pentose sugars and R5P, which are utilized for anabolic processes such as the synthesis of purine nucleotides and aromatic amino acids. Alternatively, the pentose sugars may feed back into glycolysis at steps where F6P and dihydroxyacetone phosphate (DHAP) are formed. Decreased PPP metabolites in AD brain suggests that AD brain is susceptible to oxidative stress and cellular injury. The decreased PPP metabolites and NADPH suggest a decreased ability to synthesize nucleotides necessary for maintaining the integrity of DNA, amino acids for neurotransmitter synthesis and fatty acids for components of myelin, which may further exacerbate neuronal degeneration. Decreased flux through glycolysis and PPP is expected to lead to decreased adenosine triphosphate (ATP), nicotinamide adenine dinucleotide (NADH) and NADPH, exacerbating cellular energy deficits.

Glycolysis is regulated primarily by three key enzymes, hexokinase (HK), phosphofructokinase-1 (PFK1) and pyruvate kinase (PK), as well as allosteric factors including pH, adenosine monophosphate (AMP) and ATP. An et al. utilized the ratio of the glycolytic amino acids serine, glycine, and alanine to the glucose concentrations in the inferior temporal gyrus and middle frontal gyrus and found that the activities of the three rate-limiting enzymes HK, PFK1, and PK were significantly reduced in AD, correlating with more severe AD pathology (28). We observed a distinct difference between AD and CTRL brain in the profile of glucose-derived metabolites. In AD brain, all nominally correlated glucose-derived metabolites were positively correlated, suggesting that G6P fluxes through glycolysis and the PPP relatively evenly. And in both cohorts, G6P, pyruvate and R5P are all positively correlated. But in CTRL brain, the bloc of glycolytic metabolites DHAP through PEP are positively correlated with each other but negatively correlated with G6P, pyruvate and R5P. Although this differential flux through glycolysis remains to be explained, it is possible that in CTRL brain higher levels of G6P lead to higher levels of ATP, which allosterically inhibits PFK1, and vice versa, resulting in an inverse correlation between G6P and downstream glycolytic intermediates. Alternatively, or additionally, energy-replete CTRL brain may favor use of F6P and G3P to synthesize R5P as substrate for production of other biomolecules. In relatively ATP-deprived AD brain, the opposite of the latter pathway may occur, with R5P reentering glycolysis to produce ATP and pyruvate for further ATP production at the expense of production of important building block molecules.

**Figure 4.**
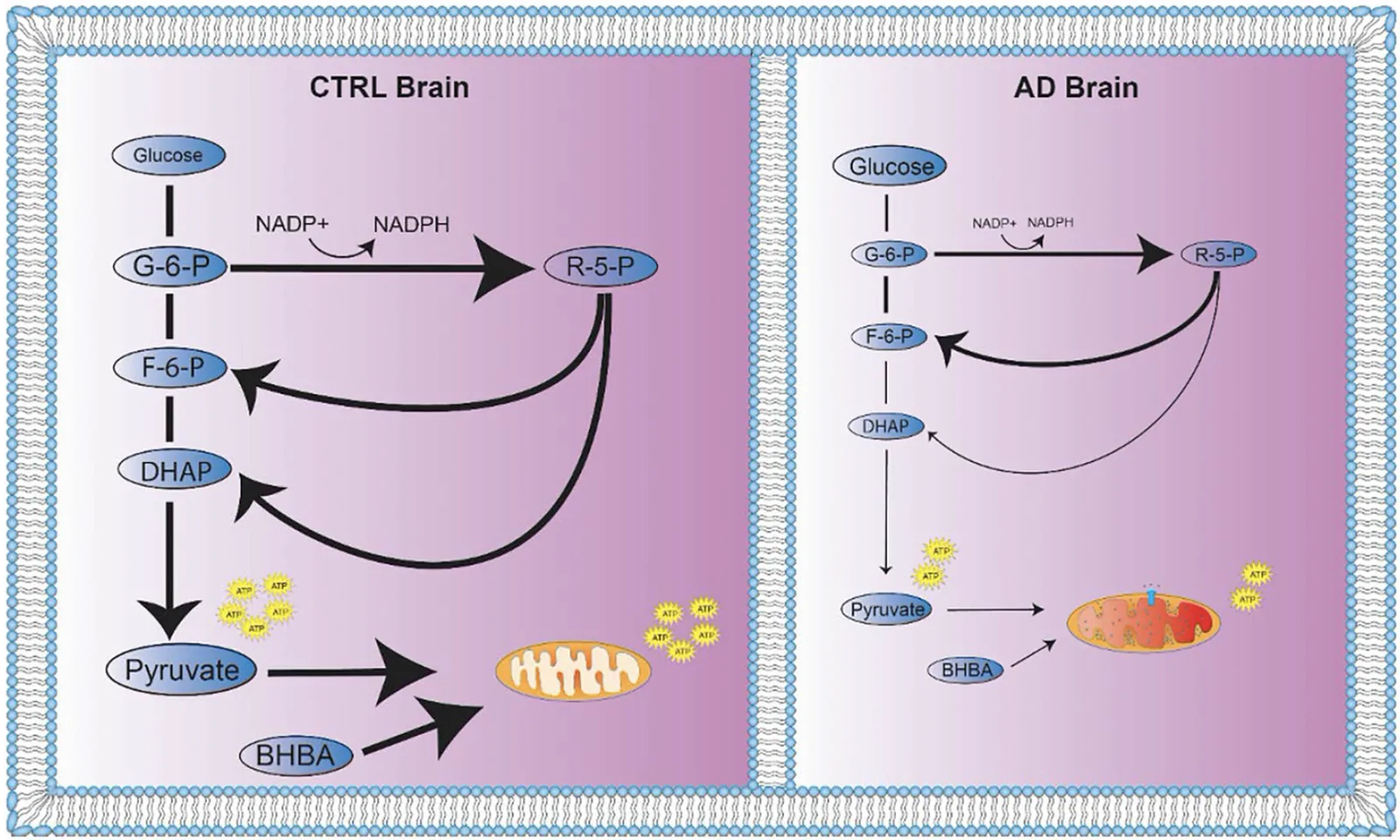
Global energy metabolism deficit in AD brain. Flux through energy metabolism pathways is indicated by solid black arrows where the thickness of the arrow represents greater relative flux. Greater relative concentrations of metabolites are represented by larger size of molecule names. (A) In CTRL brain, the relative concentrations of glucose derived energy metabolites and BHBA were increased. Energy metabolism flux in CTRL brain is distributed through glycolysis, PPP, and oxidative phosphorylation as needed for energy, redox homeostasis and molecular building block requirements. When blood glucose levels are low, or energy metabolism shifts towards ketone body production, BHBA is taken up by brain as an alternative energy source. (B) In AD brain, glucose derived energy metabolites and BHBA were decreased. Glycolytic and PPP metabolite correlations indicate strong correlations of G6P with F6P and DHAP, suggesting a possible recycling of PPP metabolites to replenish NADPH for neutralization of oxidative species. In the setting of impaired mitochondrial function, pyruvate is preferentially reduced to lactate to regenerate NAD+ to continue glycolysis for ATP production. BHBA levels are decreased, contributing to the energy deficit

Larger and more detailed studies of CTRL and AD brain are required to understand differences in glucose metabolism. However, a hypothetical model of relative abundance of ATP in CTRL brain resulting from greater flux through glycolysis plus greater access to BHBA suggests the possibility of a more robust and selective process of glucose metabolism in CTRL compared to AD brain (Figure 4).

We focused our study on glycolysis and the PPP as these are primordial energy metabolism pathways whose origins likely precede life itself (29). Arguably, every physiological function in living cells is dependent on these pathways. But our previous work confirmed that in erythrocytes there is wide biological variability in flux through glycolysis and the PPP that is strongly genetically determined (16, 30–32). In erythrocytes, concentrations of glycolytic intermediates are inherited en bloc (16), resembling our observations in brain of positive correlations among glycolytic intermediates. Although our study does not address heritability in brain energy metabolism, our findings of differential flux through glycolysis and the PPP in an en bloc pattern would be consistent with a hypothesis of a genetic contribution to brain energy metabolism, and potentially, susceptibility to AD.

Limitations of this pilot study include a small sample size and the inability to differentiate between gray matter, white matter, neurons or glia.

## Conclusion

Our findings indicate a uniform decrease in the relative concentration of glucose-derived metabolites combined with decreased relative concentrations of BHBA in the pre-frontal cortex of AD compared to CTRL brain. These observations suggest that global energy metabolism deficits may contribute to AD pathology. Further studies investigating the relationship between systemic energy metabolism and AD will advance the understanding of AD pathogenesis.

## Supplementary Material

Supplemental Information

## Figures and Tables

**Table 1.** Demographic data and neuropathologic diagnosis of subjects

Identifier	Gender	Age (years)	PMI (hours:mins)	Diagnosis
AD1	F	63	3:18	A3B3C3
AD2	F	89	3:22	A3B3C3
AD3	F	89	4:15	A3B3C3
AD4	F	86	4:25	A3B3C3
AD5	F	72	4:26	A3B3C3
AD6	M	79	5:18	A3B3C3
AD7	F	73	9:10	A3B3C3
AD8	F	87	11:45	A3B3C3
AD9	M	80	15:25	A3B3C3
AD10	F	88	19:30	A3B3C3
CTRL 1	F	86	3:30	Hippocampal sclerosis
CTRL 2	F	41	4:15	Leukodystrophy
CTRL 3	M	> 90	5:18	Argyrophilic grain disease
CTRL 4	F	86	6:22	Glioblastoma
CTRL 5	F	86	11:40	Hippocampal sclerosis
CTRL 6	M	> 90	15:24	Normal adult brain
CTRL 7	F	70	5:05	Normal adult brain
CTRL 8	F	80	3:00	Parkinson plus
CTRL 9	F	69	6:00	Amyotrophic lateral sclerosis
CTRL 10	F	81	19:15	Hippocampal sclerosis
