# Supplementary material for: Global Energy Metabolism Deficit in Alzheimer Disease Brain
Source: J Prev Alzheimers Dis. Author manuscript; Available in PMC 2026 Sep 12. (PMC13570669; doi:10.14283/jpad.2023.91)
Supplement: Supplementary file 1 [file NIHMS2208738-supplement-Supplemental_Information.docx]

**Appendices (supplemental tables)**

**Supplemental Files**

**Supplementary Tables**

**Supplementary Table 1: Standards used in LC-MS methods.**

| Method | Standards Used |
| --- | --- |
| RPLC negative mode | **Instrument performance/QC standards:** d7-glucose, d3-methionine, d3-leucine, d8-phenylalanine, d5-tryptophan, bromophenylalanine, d15-octanoic acid, d19-decanoic acid, d27-tetradecanoic acid, d35-octadecanoic acid, d2-eicosanoic acid |
|  | **Recovery/process assessment standards:** tridecanoic acid, chlorophenylalanine |
| RPLC positive mode | **Instrument performance/QC standards:** d7-glucose, d3-methionine, d3-leucine, d8-phenylalanine, d5-tryptophan, bromophenylalanine, d4-tyrosine, d5-indole acetic acid, d5-hippuric acid, amitriptyline, d9-progesterone, d4-dioctylphthalate |
|  | **Recovery/process assessment standards:** d6-cholesterol, chlorophenylalanine |
| HILIC LC | **Instrument performance/QC standards:** d35-octadecanoic acid, d5-indole acetic acid, bromophenylalanine, d5-tryptophan, d4-tyrosine, d3-serine, d3-aspartic acid, d7-ornithine, d4-lysine |
|  | **Recovery/process assessment standards:** fluorophenylglycine, chlorophenylalanine |

**Supplementary Table 2: Quality control results.**

| QC Sample | Measurement | Median RSD |
| --- | --- | --- |
| Internal standards | Instrument variability | 3% |
| Endogenous biochemicals | Total process variability | 9% |

**Supplementary Table 3: Mass spectrometric parameters.**

|  | RPLC Positive Hydrophilic | RPLC Positive Hydrophobic | RPLC Negative | HILIC |
| --- | --- | --- | --- | --- |
| Spray voltage (V) | 3300 | 4000 | 4200 | 3000 |
| Mass range (m/z) | 80-1000 | 70-1000 | 110-1000 | 80-1000 |
| Sheath gas (au) | 70 | 70 | 35 | 60 |
| Auxiliary gas (au) | 15 | 35 | 35 | 60 |
| Source temp (°C) | 300 | 300 | 400 | 300 |
| Ion transfer tube temp (°C) | 250 | 250 | 320 | 250 |
| Norm. collision energy (au) | 52, 65, 78 | 52, 65, 78 | 52, 65, 78 | 48, 60, 72 |
| MS AGC target (au) | 1e6 | 1e6 | 1e6 | 1e6 |
| MS max fill time (ms) | 60 | 60 | 60 | 60 |
| MS^n^ target (au) | 2e5 | 2e5 | 2e5 | 2e5 |
| MS^n^ max fill time (ms) | 120 | 120 | 120 | 120 |
| MS^n^ isolation window | 3 | 3 | 3 | 3 |
| MS^n^ dynamic exclusion time (s) | 3 | 3 | 3 | 3 |
| S-lens RF level | 40 | 40 | 50 | 25 |

**Supplementary Table 4: Statistically significant metabolites in AD brain.**

| **Metabolite** | **Sub Pathway** | **p-value** | **Fold Change** |
| --- | --- | --- | --- |
|  |  |  |  |
| N-acetylaspartate (NAA) | Alanine and Aspartate | 0.026 | -0.321 |
| N-acetylalanine | Alanine and Aspartate | 0.038 | -0.342 |
| N-acetylneuraminate | Aminosugar Metabolism | 0.014 | -0.452 |
| N-stearoyl-sphingadienine (d18:2/18:0)* | Ceramides | 0.038 | -0.406 |
| sphingomyelin (d18:0/18:0, d19:0/17:0)* | Dihydrosphingomyelins | 0.004 | -0.448 |
| sphingomyelin (d18:0/20:0, d16:0/22:0)* | Dihydrosphingomyelins | 0.005 | -0.631 |
| glycylleucine | Dipeptide | 0.013 | -2.489 |
| glycylvaline | Dipeptide | 0.015 | -1.810 |
| valylglycine | Dipeptide | 0.047 | -2.777 |
| arachidonoyl ethanolamide | Endocannabinoid | 0.005 | -0.534 |
| stearoyl ethanolamide | Endocannabinoid | 0.019 | -0.389 |
| eicosenoylcarnitine (C20:1)* | Fatty Acid Metabolism | 0.041 | -0.513 |
| (16 or 17)-methylstearate (a19:0 or i19:0) | Fatty Acid, Branched | 0.028 | -0.900 |
| (14 or 15)-methylpalmitate (a17:0 or i17:0) | Fatty Acid, Branched | 0.044 | -0.729 |
| 3-hydroxyhexanoate | Fatty Acid, Monohydroxy | 0.031 | -0.468 |
| gluconate | Food Component/Plant | 0.001 | -1.145 |
| galactose 1-phosphate | Fructose, Mannose and Galactose | 0.020 | -1.557 |
| N-acetylglutamate | Glutamate Metabolism | 0.002 | -1.012 |
| fructose-6-phosphate | Glycolysis and Gluconeogenesis | 0.029 | -1.529 |
| pyruvate | Glycolysis and Gluconeogenesis | 0.032 | -1.222 |
| N-acetylhistidine | Histidine Metabolism | 0.006 | -1.798 |
| 1-methylhistamine | Histidine Metabolism | 0.032 | 1.094 |
| inositol 1-phosphate (I1P) | Inositol Metabolism | 0.048 | -1.014 |
| 3-hydroxybutyrate (BHBA) | Ketone Bodies | 0.006 | -1.709 |
| tiglylcarnitine (C5:1-DC) | Leucine, Isoleucine and Valine | 0.029 | -1.091 |
| N-acetylvaline | Leucine, Isoleucine and Valine | 0.035 | -0.220 |
| 3-hydroxyisobutyrate | Leucine, Isoleucine and Valine | 0.039 | -0.609 |
| stearate (18:0) | Long Chain Fatty Acid | 0.025 | -0.727 |
| lysine | Lysine Metabolism | 0.048 | -0.365 |
| 1-oleoyl-GPC (18:1) | Lysophospholipid | 0.012 | -1.235 |
| 1-arachidonoyl-GPE (20:4n6)* | Lysophospholipid | 0.018 | -1.365 |
| 2-palmitoyl-GPC (16:0)* | Lysophospholipid | 0.019 | -1.501 |
| 1-arachidonoyl-GPI (20:4)* | Lysophospholipid | 0.035 | -1.024 |
| N-acetylmethionine | Methionine, Cysteine, SAM | 0.013 | -0.486 |
| N-formylmethionine | Methionine, Cysteine, SAM | 0.021 | -0.576 |
| sedoheptulose | Pentose Metabolism | 0.032 | -0.607 |
| ribulose/xylulose | Pentose Metabolism | 0.042 | -0.717 |
| sedoheptulose-7-phosphate | Pentose Phosphate Pathway | 0.007 | -0.719 |
| 1-myristoyl-2-arachidonoyl-GPC (14:0/20:4)* | Phosphatidylcholine (PC) | 0.006 | -0.491 |
| 1-linoleoyl-2-arachidonoyl-GPC (18:2/20:4n6)* | Phosphatidylcholine (PC) | 0.010 | -0.671 |
| 1-stearoyl-2-arachidonoyl-GPC (18:0/20:4) | Phosphatidylcholine (PC) | 0.013 | -0.182 |
| 1,2-distearoyl-GPC (18:0/18:0) | Phosphatidylcholine (PC) | 0.026 | -0.657 |
| 1-palmitoyl-2-arachidonoyl-GPC (16:0/20:4n6) | Phosphatidylcholine (PC) | 0.034 | -0.219 |
| 1-oleoyl-2-docosahexaenoyl-GPC (18:1/22:6)* | Phosphatidylcholine (PC) | 0.034 | -0.251 |
| 1-palmitoyl-2-alpha-linolenoyl-GPC (16:0/18:3n3)* | Phosphatidylcholine (PC) | 0.039 | -0.587 |
| 1-stearoyl-2-arachidonoyl-GPE (18:0/20:4) | Phosphatidylethanolamine (PE) | 0.003 | -0.402 |
| 1-palmitoyl-2-oleoyl-GPE (16:0/18:1) | Phosphatidylethanolamine (PE) | 0.010 | -0.217 |
| 1-oleoyl-2-docosahexaenoyl-GPE (18:1/22:6)* | Phosphatidylethanolamine (PE) | 0.010 | -0.311 |
| 1-stearoyl-2-oleoyl-GPE (18:0/18:1) | Phosphatidylethanolamine (PE) | 0.013 | -0.487 |
| 1-palmitoyl-2-arachidonoyl-GPE (16:0/20:4)* | Phosphatidylethanolamine (PE) | 0.039 | -0.197 |
| 1-oleoyl-2-linoleoyl-GPE (18:1/18:2)* | Phosphatidylethanolamine (PE) | 0.042 | -0.520 |
| phosphoethanolamine | Phospholipid Metabolism | 0.003 | -0.497 |
| 1-(1-enyl-stearoyl)-2-arachidonoyl-GPE (P-18:0/20:4)* | Plasmalogen | 0.008 | -0.212 |
| arachidonate (20:4n6) | Polyunsaturated Fatty Acid | 0.021 | -1.318 |
| docosahexaenoate (DHA; 22:6n3) | Polyunsaturated Fatty Acid | 0.023 | -1.303 |
| adrenate (22:4n6) | Polyunsaturated Fatty Acid | 0.035 | -1.397 |
| docosapentaenoate (n6 DPA; 22:5n6) | Polyunsaturated Fatty Acid | 0.042 | -1.200 |
| xanthosine | Purine Metabolism | 0.009 | -0.490 |
| xanthine | Purine Metabolism | 0.021 | -0.335 |
| guanosine | Purine Metabolism | 0.046 | -0.615 |
| 2'-deoxyuridine | Pyrimidine Metabolism, Uracil | 0.040 | -0.507 |
| flavin adenine dinucleotide (FAD) | Riboflavin Metabolism | 0.026 | -0.497 |
| sphingomyelin (d18:1/20:1, d18:2/20:0)* | Sphingomyelins | 0.003 | -0.498 |
| sphingomyelin (d18:1/20:0, d16:1/22:0)* | Sphingomyelins | 0.037 | -0.467 |
| alpha-tocopherol | Tocopherol Metabolism | 0.031 | -0.451 |

***:** Indicates compounds that not officially confirmed based on a standard, however Metabolon is confident in their identity.
